# Quinolinic acid is associated with cognitive deficits in schizophrenia but not major depressive disorder

**DOI:** 10.1038/s41598-021-89335-9

**Published:** 2021-05-11

**Authors:** Flurin Cathomas, Karoline Guetter, Erich Seifritz, Federica Klaus, Stefan Kaiser

**Affiliations:** 1 Department of Psychiatry, Psychotherapy and Psychosomatics, Psychiatric Hospital, University of Zurich, Lenggstrasse 31, 8032 Zürich, Switzerland; 2 Fishberg Department of Neuroscience, Friedman Brain Institute, Icahn School of Medicine at Mount Sinai, Mount Sinai, NY USA; 3 Department of Psychiatry, University of California San Diego, San Diego, USA; 4 Neuroscience Center Zurich, ETH Zurich and University of Zurich, 8057 Zurich, Switzerland; 5 Division of Adult Psychiatry, Department of Psychiatry, Geneva University Hospitals, Chemin du Petit-Bel-Air, 1225 Chêne-Bourg, Switzerland

**Keywords:** Cognitive control, Neuroscience, Neuroimmunology

## Abstract

Tryptophan and its catabolites (TRYCATs) have been suggested to link peripheral immune system activation and central neurotransmitter abnormalities with relevance to the etio-pathophysiology of schizophrenia (SZ) and major depressive disorder (MDD). The relationship to different psychopathological dimensions within these disorders however remains to be elucidated. We thus investigated potential group differences of tryptophan, kynurenine, kynurenic acid, 3-hydroxy kynurenine and quinolinic acid in the plasma of 19 healthy controls (HC), 45 patients with SZ and 43 patients with MDD and correlated plasma proteins with the “motivation and pleasure” dimension and cognition. After correcting for the covariates age, sex, body mass index, smoking and medication, patients with MDD showed lower kynurenine and 3-hydroxy kynurenine levels compared to HC. Quinolinic acid correlated negatively with composite cognitive score in patients with SZ, indicating that more severe cognitive impairments were associated with increased plasma levels of quinolinic acid. No correlations were found in patients with MDD. These results indicate that MDD and SZ are associated with dysregulation of the kynurenine pathway. Quinolinic acid might be specifically implicated in the pathophysiology of cognitive deficits in patients with SZ. Further studies are needed to determine whether TRYCATs are causally involved in the etiology of these neuropsychiatric disorders.

## Introduction

Schizophrenia (SZ) and major depressive disorder (MDD) are prevalent neuropsychiatric disorders with high socioeconomic and individual burden^1,2^. Despite their significant contribution to the global burden of disabilities^3^ our knowledge of the underlying etio-pathophysiological mechanisms are sparse and as a result, treatment options remain limited^4^.

Both SZ and MDD are heterogeneous syndromes consisting of several symptom dimensions^5,6^. While for a long time considered as separate disorders, there is increasing evidence from clinical psychopathology, neuroimaging and genetic studies that there are both different and overlapping features that characterize these disorders^7^. Therefore, detailed psychopathological characterization and comparison of symptom dimensions with potential biological markers is important. Motivational deficits are symptoms of several psychiatric disorders, including SZ and MDD^8^. Apathy, a reduction of motivation and goal-directed behaviours, has been shown to be prevalent in both patients with MDD^9^ and SZ^10^. Further, cognitive dysfunction has been consistently described in both patients with SZ and MDD^11,12^.

There is increasing evidence that the immune system plays a major role in neuropsychiatric disorders, including being causally involved in the pathogenesis of the above discussed symptom dimensions^13,14^. One candidate pathway linking peripheral immune system activation and central neurotransmitter abnormalities involves tryptophan (TRP) and its catabolites (TRYCATs)^15^. The essential amino acid TRP is catabolized into several bioactive molecules, including serotonin and products of the kynurenine pathway^15^. Two enzymes are mainly involved in catabolizing tryptophan to kynurenines: Indoleamine 2,3-dioxygenase (IDO) (which can be indirectly assessed by the tryptophan / kynurenine (KYN) ratio, see below), which in the periphery is mainly present in myeloid cells, and the predominantly hepatic enzyme tryptophan 2,3-dioxygenase (TDO). Upon activation by pro-inflammatory cytokines such as interleukin (IL)-6, IL-1β and interferon-γ^16,17^, TRP is catabolized to kynurenine. Kynurenine can then be catabolized along two pathways: Kynurenine aminotransferases produce kynureninc acid (KYNA) and kynurenine monooxygenase (KMO) converts KYN to 3-hydroxykynurenine (3-OHK), which is further catabolized to quinolinic acid (QUIN). Importantly, KYNA, 3-OHK and QUIN were shown to exhibit neuroactive properties, including affecting glutamatergic, dopaminergic and nicotinergic neurotransmission^18^.

Evidence for a causal role of kynurenine pathway dysregulation leading to behavioural alterations in several neuropsychiatric disorders comes from animal models, in which it was shown that pharmacologically blocking IDO prevents depression-like behaviours induced by immune stimulation using the bacterial endotoxin Lipopolysaccharide (LPS) or social defeat stress^19,20^.

Several studies have so far investigated group differences in TRYCATs in the circulation of patients with SZ and MDD, respectively, but have reported inconclusive findings. Recent meta-analyses have shown that KYNA and KYN blood levels were lower in patients with MDD in comparison to healthy controls (HC), while QUIN levels did not differ between the two groups^21,22^. In SZ, the findings are even more inconclusive: A recent meta-analysis has not found any differences in peripheral KYNA^23^. One large study has found decreased plasma concentrations of 3-OHK in patients with SZ compared to HC, but not other TRYCATs^24^. However, most studies up to date have focused on differences between diagnostic groups rather than correlating TRYCATs with various symptom dimensions across different diagnoses.

The overall hypothesis of the present study was that both MDD and SZ are associated with dysregulation of the kynurenine pathway. Specifically, based on the above discussed literature, we hypothesized that compared to HC, patients with MDD would display lower levels of KYN and KYNA, and patients with SZ would show lower levels of 3-OHK. Based on findings from pre-clinical animal models, we further hypothesized that in both patients with MDD and SZ, motivational deficits would correlate with the KYN/TRP ratio, as an indicator of IDO activity and the neurotoxic TRYCAT QUIN would be associated with cognitive deficits. Thus, we first investigated group differences in TRYCATs in the plasma of HC, SZ and MDD subjects and then correlated TRYCAT levels with the psychopathological dimensions “motivation and pleasure” and cognition.

## Methods

### Participants

45 patients meeting the DSM-IV (American Psychiatric Association, 2000) criteria for SZ, 44 patients with MDD and 19 HC were recruited. One patient with MDD was excluded from the study because of technical problems. Patients with other SZ spectrum disorder were not included. More patients (SZ and MDD) than HC were recruited to have adequate power for the correlational analyses. Patients were recruited from outpatient and inpatient units of the Psychiatric Hospital of the University of Zurich and affiliated institutions. Diagnoses were confirmed by conducting the Mini-International Neuropsychiatric Interview^25^. All patients were clinically stable and under a stable dose of medication for at least two weeks prior to testing. Inpatients were at the end of their hospitalization and engaged in a multimodal therapy program and activities outside the hospital. The average duration of hospitalization for patients with SZ and MDD in Swiss psychiatric hospitals is longer than in most other countries, so the majority of inpatients would have been treated as outpatients in other health care systems. HC were recruited from the community via advertisement. All participants gave written informed consent and the project was approved by the Ethics Committee of the Canton of Zurich. Assessment of psychopathology and cognition, and blood draw were performed on the same day and in accordance with relevant guidelines and regulations. The inclusion age was between 18 and 65 years.

Exclusion criteria were similar to our previous studies^26–28^. We excluded patients with any other than the above mentioned DSM-IV Axis I disorders, benzodiazepines (except less than 1 mg lorazepam per day) and acute psychotic symptoms. Participants with any alcohol use disorder based on lifetime criteria and participants with a current abuse or dependency of cannabis or any other substance abuse were excluded. HC were excluded if any psychiatric diagnosis was present in the structured Mini-International Neuropsychiatric Interview. In both groups, participants were excluded, if they had a history of head-injury or any autoimmune or chronic inflammatory disorder or if they took any pain-medication or anti-inflammatory drugs at least one week prior to testing (assessed by detailed questionnaire and medical records where available). Furthermore, participants were not included in the study if they had a history of any known acute inflammation two weeks prior to testing. Chlorpromazine equivalents were calculated according to^29^ and imipramine equivalents according to^30^.

### Assessment of psychopathology and cognition

Motivation was assessed using the “motivation and pleasure” dimension (sum score of the subscales anhedonia (items 1–3), asociality (items 5, 6) and avolition (items 7, 8)) of the Brief Negative Symptom Scale (BNSS)^31,32^. Cognition was assessed with the Brief Neurocognitive Assessment (BNA)^33^. With the BNA, a cognitive score is computed for each participant by combining results from the Letter-Number-Span Test and the Symbol Coding Test. The BNA has been shown to be highly correlated with the MATRICS Consensus Cognitive Battery (MCCB)^34^ and has similarly good validity criteria^35^. Positive symptoms were assessed with the Positive and Negative Syndrome Scale (PANSS)^36,37^. Depressive symptoms were assessed using the Beck Depression Inventory (BDI)^38^ and Calgary Depression Scale for Schizophrenia (CDSS)^39^. Global level of functioning was assessed with the adapted version of the Personal and Social Performance Scale (PSP)^40,41^.

### Blood draw and processing

Blood was drawn between 8 and 10 am on the day of the psychopathological assessment. Study participants were instructed to fasten for at least 8 h prior to the blood draw which was verified by a questionnaire on the day of testing. To obtain plasma, blood was drawn into ethylenediaminetetraacetic acid (EDTA) tubes (Sarstedt, Switzerland), centrifuged for 15 min at 1500×*g* and plasma was frozen at − 80 °C.

### TRYCATs

TRYCATs were measured by Brains Online, LLC^42,43^. Plasma samples were prepared for analysis of KYN, KYNA, 3-OH-Kynurenine 3-OHK, and TRP by mixing 5 µL of sample with 40 µL of internal standard solution (containing KYN-D4, KYNA-D5, 3-OH-KYN-13C6 and TRP-D5). Plasma samples were prepared for analysis of Quinolinic acid (QUIN) by mixing 10 µL of sample with 40 µL of internal standard solution (containing QUIN-D3). All samples were incubated at room temperature and centrifuged at 13,000 rpm for 5 min. The resultant supernatant was used for liquid tandem mass spectrometry (LC–MS/MS) analysis. Calibrators, QC’s and blanks were prepared following the same procedures using blank plasma. Concentrations of KYN, KYNA, 3-OH-KYN, AA and TRP were determined by high performance liquid chromatography (HPLC) with tandem mass spectrometry (MS/MS) detection. Of each sample, an aliquot was injected onto the HPLC column by an automated sample injector (SIL10-AD, Shimadzu, Japan). Chromatographic separation was performed on a reversed phase analytical column (150 × 2.1 mm, 3.0 µm) held at a temperature of 25 °C. Components were separated using a two-step linear gradient of ultrapurified H_2_O with 0.1% formic acid (FA) and acetonitrile with 0.1% FA at a flow rate of 0.2 mL/min 1, 2. The MS analyses were performed using an API 4000 MS/MS system, equipped with a Turbo Ion Spray interface (both from Sciex). The instrument was operated in multiple-reaction-monitoring (MRM) mode. The acquisitions were performed in positive ionization mode, with optimized settings for the analytes. Concentrations of QUIN were determined by HPLC with tandem mass spectrometry (MS/MS) detection. QUIN analysis was carried out in a single analyte assay. Chromatographic separation was performed on a reversed phase analytical column (100 × 3.0 mm, 2.5 µm) held at a temperature of 25 °C. Component was separated using a linear gradient of ultrapurified H_2_O with 0.2% tri-fluoric acid (TFA) and acetonitrile with 0.2% TFA (flow rate 0.3 mL/min) 2. The MS analyses were performed using an API 4000 MS/MS system, equipped with a Turbo Ion Spray interface (both from Sciex). The instrument was operated in multiple-reaction-monitoring mode. The acquisitions were performed in positive ionization mode, with optimized settings for QUIN. Suitable in-run calibration curves were fitted using weighted (1/x) regression, and the sample concentrations were determined using these calibration curves. Accuracy was verified by quality control samples after each sample series. Data were acquired and processed using the Analyst data system (software version 1.6.2, Sciex). One QUIN sample from a HC had a value below limit of quantification, otherwise, all values were above lower limit of detection and passed quality control.

### Data analysis

First, normal distribution was tested using the Shapiro–Wilk test. Non-normally distributed data were log-transformed. If normal distribution was achieved, parametric tests were used. If normal distribution was not achieved, parametric tests were performed for log-transformed values. Comparisons between HC, patients with SZ and patients with MDD were performed using one-way ANOVAs with Tukey’s test for post-hoc comparisons (for normal distributed data) and Kruskal–Wallis test with Dunn’s test for post-hoc comparisons (for non-normally distributed data). General linear models (post-hoc test: least significant difference) were used to test for the effects of covariates [(age, sex, BMI, smoking and medication (chlorpromazine equivalents, imipramine equivalents, lorazepam medication)]. For correlations between TRYCATs and symptom dimensions Pearson correlation coefficients (normal distribution) or Spearman correlation coefficients (non-normally distributed data) were calculated. Partial correlations were calculated when controlling for covariates (age, sex, BMI, smoking, disease severity (i.e. illness onset and duration), medication (chlorpromazine equivalents, imipramine equivalents, lorazepam medication) and total years of education) using partial correlations. No correlations were calculated for the HC group, based on the low level of psychopathological symptoms. The level of statistical significance was set at p < 0.05. Statistical analyses were computed with SPSS version 25 (IBM Corp., SPSS Inc., Chicago IL, USA) and GraphPad Prism software 8 (GraphPad Software Inc.).

### Ethics approval and consent to participate

All participants gave written informed consent and the project was approved by the Ethics Committee of the Canton of Zurich.

## Results

Sociodemographic data of the sample and statistics are summarized in Table 1. While there were no differences in sex between HC and patient groups, there were more female participants in the MDD group compared to the SZ group. Patients with SZ had experienced on average 5.3 psychotic episodes, while patients with MDD had 3.6 depressive episode. Body Mass Index (BMI) was higher in patients with SZ than HC or patients with MDD, with no difference between MDD and HC. Patients with SZ and MDD had similar motivational deficits [assessed by the “motivation and pleasure” dimension (BNSS)] compared to HC, but patients with MDD showed higher levels of depressive symptoms. Compared to HC and patients with MDD, patients with SZ showed significant deficits in the cognitive dimension (assessed by the BNA), while there were no differences between patients with MDD and HC. Patients with SZ showed more positive symptoms (assessed by the PANSS positive symptom subscale) than patients with MDD. Both, patients with SZ and MDD showed similar levels of functional impairment compared to HC, indicated by lower levels on the PSP. Patients with SZ showed fewer years of education compared to patients with MDD and HC.

**Table 1:** Sociodemographic and clinical data.

	HC (n = 19)	SZ (n = 45)	MDD (n = 43)	Statistics		
				Test statistics		Post-hoc
Age (years)	32.53 ± 9.45	34.00 ± 10.47	35.79 ± 10.88	KWS = 1.37	*p* = 0.503^2^	
Sex (male/female)	9/10	31/14	17/26	*X*^*2*^ = 7.94	***p = 0.019***^3^	*p* = 0.312^a^ *p* = 1.000^b^ ***p = 0.018***^c^
Smoking (no/yes)	13/6	16/29	29/14	*X*^*2*^ = 10.887	**p = 0.004**^3^	***p = 0.048***^a^ *p* = 1.000^b^ ***p = 0.009***^c^
Education (years)^d^	14.39 ± 2.23	12.26 ± 3.88	14.57 ± 3.17	*KWS* = 15.25	***p < 0.001***^2^	***p = 0.013***^a^ *p* > 0.999^b^ ***p = 0.001***^c^
Body mass index	23.40 ± 4.32	26.40 ± 4.66	22.88 ± 4.30	KWS = 15.44	***p < 0.001***^2^	***p = 0.025***^a^ *p* > 0.999^b^ ***p < 0.001***^c^
Number of psychotic episodes	0	5.31 ± 5.14	0	KWS = 93.33	***p < 0.001***^2^	***p < 0.001***^a^ *p* > 0.999^b^ ***p < 0.001***^c^
Number of depressive episodes	0	0.09 ± 0.29	3.60 ± 3.19	KWS = 86.75	***p < 0.001***^2^	*p* > 0.999^a^ ***p < 0.001***^b^ ***p < 0.001***^c^
Age at illness onset (years)	–	23.91 ± 6.88	28.77 ± 10.89	U = 722	**p = 0.041**^4^	
Illness duration (months)	–	121.07 ± 101.28	84.26 ± 95.46	U = 736	p = 0.054^4^	
Antipsychotic medication (yes/no)	–	44/1	2/41	*X*^*2*^ = 76.44	***p < 0.001***^3^	
Chlorpromazine equivalents (mg/day)	–	514.67 ± 444.82	2.34 ± 11.31	U = 23.5	***p < 0.001***^4^	
Antidepressant medication (yes/no)	–	6/39	33/10	*X*^*2*^ = 10.89	***p < 0.001***^3^	
Imipramine equivalents	–	21.43 ± 60.86	106.10 ± 93.57	U = 376.5	***p < 0.001***^4^	
Lorazepam (yes/no)	–	3/42	3/40	*X*^*2*^ = 0.003	***p = 0.954***^3^	
CDSS (total)	–	3.00 ± 3.65	12.33 ± 4.95	U = 118.5	***p < 0.001***^4^	
BDI (total)	–	14.16 ± 9.84	26.07 ± 12.54	U = 433	***p < 0.001***^4^	
PANSS positive factor	–	5.96 ± 2.70	4.37 ± 1.07	U = 574	***p < 0.001***^4^	
Motivation and pleasure dimension (BNSS)	0.74 ± 1.10	18.40 ± 9.46	20.58 ± 7.19	KWS = 44.97	***p < 0.001***^2^	***p < 0.001***^a^ ***p < 0.001***^b^ *p* > 0.999^c^
Cognitive dimension (BNA)	0.00 ± 0.68	− 0.88 ± 0.70	− 0.12 ± 0.85	F(2, 100) = 13.83	***p < 0.001***^1^	***p < 0.001***^a^ *p* = 0.848^b^ ***p < 0.001***^c^
Global functioning (PSP total)	97.05 ± 4.70	51.78 ± 14.42	57.26 ± 16.77	KWS = 47.67	***p < 0.001***^2^	***p < 0.001***^a^ ***p < 0.001***^b^ *p* = 0.580^c^

Statistics: ^1^One-way analysis of variance (ANOVA), ^2^Kruskal-Wallis test, ^3^Chi-square test, ^4^Mann-Whitney U test. Post-hoc. Tukey’s test for post-hoc comparisons of ANOVAs and Dunn’s test for post-hoc comparison of Kruskal–Wallis test: ^a^HC vs. SZ, ^b^HC vs. MDD, ^c^SZ vs. MDD.* BDI* Beck Depression Inventory, *BNA* Brief Neurocognitive Assessment, *BNSS* Brief Negative Symptom Scale, *CDSS* Calgary Depression Scale for Schizophrenia, *HC* Healthy controls, *MDD* Major depressive disorder, *PANSS* Positive and Negative Syndrome Scale, *PSP* Personal and Social Performance Scale, *SZ* Schizophrenia. Data is presented as mean ± standard deviation. The level of significance was set at *p* < 0.05 (significant* p*-values are indicated in bold). ^d^Compulsory education in Switzerland is 9 years.

### 3-OHK and KYN plasma levels differ between disorders

**Figure 1:**
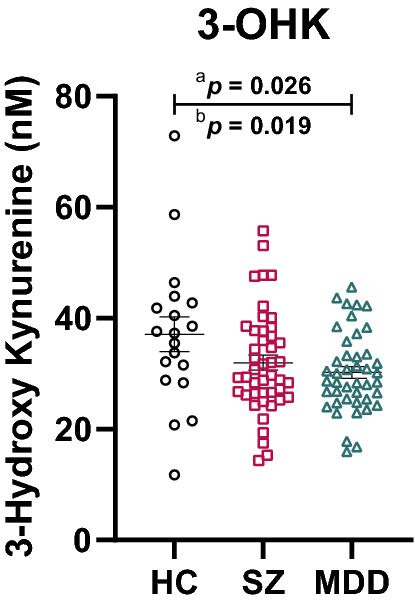
Group differences of 3-hydroxy kynurenine (3-OHK). Compared to healthy controls (HC), patients with major depressive disorder (MDD) but not schizophrenia (SZ) display a reduction in 3-OHK. ^a^Without correcting for covariates, ^b^After correction for the covariates age, sex, BMI, smoking and medication (chlorpromazine equivalents, imipramine equivalents, lorazepam medication). The level of significance was set at *p* < 0.05 (significant *p*-values are indicated in bold).

We first investigated group differences in TRYCATs (see Table 2 for detailed test statistics). There was a significant reduction of 3-OHK in patients with MDD compared to HC (uncorrected: *p* = 0.026, corrected for covariates age, sex, BMI, smoking and medication: *p* = 0.019) (Fig. 1), but no differences between SZ and HC (uncorrected: *p* = 0.122, corrected: *p* = 0.068) or SZ and MDD (uncorrected: *p* = 0.660, corrected: *p* = 0.661). There was a significant reduction of KYN specifically in patients with MDD compared to HC only after correcting for covariates (MDD vs. HC: uncorrected: *p* = 0.181, corrected: *p* = 0.016; SZ vs. HC: uncorrected: *p* = 0.427, corrected: 0.067; SZ vs. MDD: uncorrected: *p* = 0.764, corrected: *p* = 0.622). We also observed a reduction of KYNA in patients with MDD compared to HC (*p* = 0.046) but this reduction was no longer significant after introducing covariates (*p* = 0.336). No differences in KYNA were present between patients with SZ and HC (uncorrected: *p* > 0.999, corrected: *p* = 0.167) or patients with SZ and patients with MDD (uncorrected: *p* = 0.098, corrected: *p* = 0.620).

**Table 2:** Group differences in tryptophan and its catabolites between healthy controls (HC), patients with schizophrenia (SZ) or major depressive disorder (MDD).

	HC (n = 19)	SZ (n = 45)	MDD (n = 43)	Statistics					
				Test statistics (uncorrected)		Post-hoc (uncorrected)	Test statistics (corrected^6^)		Post-hoc (corrected^6^)
Tryptophan^3^ (nM)	58,539.56 ± 11,511.48	57,624.27 ± 9738.86	56,094.68 ± 9774.15	F(2,97) = 0.44	*p* = 0.648^1^		F(2, 97) = 0.313	^6^*p* = 0.732	
Kynurenine (nM)	2258.92 ± 736.61	2089.81 ± 450.33	2015.96 ± 401.99	F(2,97) = 1.59	*p* = 0.209^1^		F(2, 97) = 3.24	***p = 0.043***^**6**^	*p* = 0.067^a^ ***p = 0.016***^**b**^ *p* = 0.622^c^
Tryptophan / Kynurenine	28.16 ± 8.57	28.89 ± 8.10	28.57 ± 6.00	F(2,97) = 0.068	*p* = 0.934^1^		F(2, 97) = 1.13	*p* = 0.327^6^	
Kynurenic Acid^4^ (nM)	55.48 ± 24.56	50.55 ± 20.93	46.06 ± 28.87	KWS = 7.53	***p = 0.023***^2^	*p* > 0.999^a^ ***p = 0.046***^b^ *p* = 0.098^c^	F(2, 97) = 1.01	*p* = 0.368^6^	
3-Hydroxy Kynurenine (nM)	37.09 ± 13.58	31.99 ± 9.25	30.24 ± 7.17	F(2,97) = 3.50	***p = 0.034***^1^	*p* = 0.122^a^ ***p = 0.026***^b^ *p* = 0.660^c^	F(2, 97) = 3.11	***p = 0.049***^6^	*p* = 0.068^a^ ***p = 0.019***^**b**^ *p* = 0.661^c^
Kynurenine / 3-Hydroxy Kynurenine	62.54 ± 11.46	68.43 ± 15.91	68.99 ± 15.84	F(2,97) = 1.30	^1^*p* = 0.277		F(2, 97) = 0.12	*p* = 0.889^6^	
Kynurenic Acid / 3-Hydroxy Kynurenine^4,5^	1.48 ± 0.32	1.63 ± 0.58	1.50 ± 0.75	F(2,97) = 0.38	*p* = 0.684^1^		F(2, 97) = 0.074	*p* = 0.929^6^	
Quinolinic Acid (nM) ^4,5^	378.93 ± 478.46	341.38 ± 177.23	270.98 ± 105.48	KWS = 2.47	*p* = 0.290^2^		F(2, 97) = 0.31	*p* = 0.732^6^	
Kynurenic Acid / Quinolinic Acid^4,5^	0.23 ± 0.11	0.18 ± 0.10	0.18 ± 0.10	KWS = 4.63	*p* = 0.10^2^		F(2, 97) = 0.1	*p* = 0.908^6^	

Statistics: ^1^One-way analysis of variance, ^2^Kruskal-Wallis test, Post-hoc (Tukey’s test for post-hoc comparisons of ANOVAs and Dunn’s test for post-hoc comparison of Kruskal–Wallis tests): ^a^HC vs. SZ, ^b^HC vs. MDD, ^c^SZ vs. MDD. ^3^log10-transformation achieved normal distribution in HC, ^4^log10-transformation achieved normal distribution in patients with SZ. ^5^log10-transformation achieved normal distribution in patients with MDD. ^6^General linear model with age, sex, BMI, smoking and medication (chlorpromazine equivalents, imipramine equivalents, lorazepam medication) as covariates (post-hoc comparison: least significant difference). Data is presented as mean ± standard deviation. The level of significance was set at *p* < 0.05 (significant *p*-values are indicated in bold).

### Correlation between cognition and QUIN plasma levels in patients with SZ

Lastly, we correlated TRYCATS with the assessed psychopathological dimensions within each disorder (Table 3).

**Table 3:** Correlations between TRYCATs and psychopathological dimensions.

	Motivation and pleasure dimension (BNSS)	Cognitive symptom dimension (BNA)
Tryptophan (nM)	SZ: r(p) = − 0.043, *p* = 0.781 MDD: r(p) = 0.070, *p* = 0.657	SZ: r(p) = − 0.022, *p* = 0.887 MDD: r(p) = 0.080, *p* = 0.616
Kynurenine (nM)	SZ: r(p) = − 0.092, *p* = 0.49 MDD: r(p) = − 0.217, *p* = 0.162	SZ: r(p) = − 0.209, *p* = 0.179 MDD: r(p) = 0.052, p = 0.741
Tryptophan/kynurenine (log^1^)	SZ: r(p) = − 0.013, *p* = 0.933 MDD: r(p) = − 0.199, *p* = 0.200	SZ: r(p) = − 0.146, p = 0.349 MDD: r(p) = − 0.016, *p* = 0.919
Kynurenic acid (nM) (log^1^)	SZ: r(p) = − 0.099, *p* = 0.519 MDD: r(s) = − 0.211, *p* = 0.174	SZ: r(p) = − 0.186, *p* = 0.233 MDD: r(s) = − 0.059, *p* = 0.711
3-Hydroxy kynurenine (nM)	SZ: r(p) = − 0.025, *p* = 0.870 MDD: r(p) = − 0.202, *p* = 0.195	SZ: r(p) = − 0.089, *p* = 0.571 MDD: r(p) = − 0.126, p = 0.425
Kynurenine/3-hydroxy kynurenine	SZ: r(p) = 0.034, *p* = 0.823 MDD: r(p) = 0.057, *p* = 0.715	SZ: r(p) = − 0.061, *p* = 0.698 MDD: r(p) = 0.159, *p* = 0.316
Kynurenic acid/3-hydroxy kynurenine (log^1,2^)	SZ: r(p) = − 0.067, *p* = 0.663 MDD: r(p) = − 0.155, *p* = 0.321	SZ: r(p) = − 0.131, *p* = 0.401 MDD: r(p) = 0.019, p = 0.905
Quinolinic acid (nM) (log^1,2^)	SZ: r(p) = 0.104, *p* = 0.495 MDD: r(p) = − 0.177, *p* = 0.256	**SZ: r(p) = **− **0.324, p = 0.034** MDD: r(p) = − 0.056, *p* = 0.723
Kynurenine acid/quinolinic acid (log^1,2^)	SZ: r(p) = − 0.167, *p* = 0.273 MDD: r(p) = − 0.116, *p* = 0.459	SZ: r(p) = 0.160, *p* = 0.306 MDD: r(p) = 0.012, *p* = 0.941

Statistics: ^1^log_10_-transformation achieved normal distribution in patients with SZ, ^2^log_10_-transformation achieved normal distribution in patients with MDD. *BNA* Brief Neurocognitive Assessment, *BNSS* Brief Negative Symptom Scale, *HC* Healthy controls, *MDD* Major depressive disorder, *SZ* Schizophrenia. Data is presented as mean ± standard deviation. The level of significance was set at *p* < 0.05 (significant *p*-values are indicated in bold).

**Figure 2:**
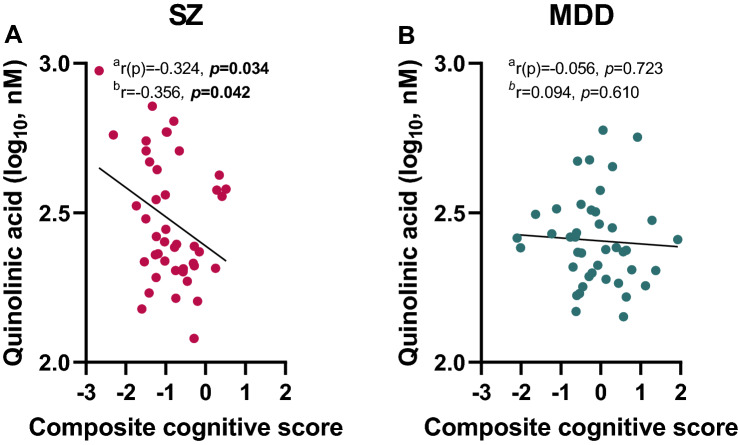
Correlations between quinolinic acid and composite cognitive score in patients with schizophrenia (SZ) and major depressive disorder (MDD). Significant negative correlations between quinolinic acid and composite cognitive score in patients with (**A**) SZ but not (**B**) MDD. Higher cognitive scores indicate better cognitive performance. ^a^Without correcting for covariates, ^b^After correction for the covariates age, sex, BMI, smoking, disease severity (i.e. illness onset and duration), medication (chlorpromazine equivalents, imipramine equivalents, lorazepam medication) and total years of education. The level of significance was set at p < 0.05 (significant p-values are indicated in bold).

Interestingly, there was a negative correlation between QUIN (log) and composite cognitive score in patients with SZ (Fig. 2A) but not MDD (Fig. 2B) (SZ: r(p) = − 0.324, *p* = 0.034; MDD: r(p) = − 0.056, *p* = 0.723), indicating that increased plasma levels of QUIN in patients with SZ are associated with increased cognitive impairment. This association remained significant after correcting for the covariates age, sex, BMI, smoking, disease severity (i.e. illness onset and duration), medication (chlorpromazine equivalents, imipramine equivalents, lorazepam medication) and total years of education (r = − 0.356, *p* = 0.042). There were no other significant correlations between TRYCATs and symptom dimensions in patients with SZ or MDD.

## Discussion

In the present study we have investigated differences in tryptophan and its catabolites between patients with SZ or MDD, and HC, and correlated the TRYCATs with the symptom dimensions “motivation and pleasure” and cognition. We show that plasma levels of 3-OHK are lower in patients with MDD compared to HC. Dimensional assessment revealed a negative correlation between plasma QUIN and composite cognitive score in patients with SZ but not in patients with MDD. In other words, cognitive impairment was related to higher plasma QUIN levels.

Several studies have investigated 3-OHK in MDD vs. HC but have not found any group differences^42–47^. Research regarding comparisons between SZ vs. HC is mixed, with some studies showing no differences^48,49^ some indicating higher^50^ and some lower^24,51^ 3-OHK levels. The biological effects of 3-OHK are complex and studies have shown both antioxidant effects potentially resulting in neuroprotection and induction of reactive oxygen species leading to neurotoxicity^52^. In addition, 3-OHK is one of the TRYCATs that is examined least frequently: a recent meta-analysis showed that out of 59 studies comparing patients with MDD vs. HC only 6 studies included 3-OHK^22^. Therefore, further studies are needed to elucidate the role of 3-OHK in the pathogenesis of MDD and SZ. After correcting for the covariates age, sex, smoking, BMI and medication, we report a reduction of KYN specifically in patients with MDD. This reduction specifically in patients with MDD compared to HC is in line with recent meta-analyses^21,22^. We additionally found a reduction of KYNA in patients with MDD. However, this reduction was no longer observed after correcting for the above mentioned covariates. While the uncorrected KYNA finding is as in line with recent meta-analyses^21,22^, it is important to note, that to our knowledge a majority of the studies that reported significant differences in KYNA in MDD vs. HC did not control for the above mentioned covariates^43,53–56^, which are known to be casually linked to a pro-inflammatory state^57,58^. Our findings can therefore be viewed in the context of the overall hypothesis that MDD and SZ are associated with a dysbalance between neurotoxic and neuroprotective tryptophan catabolites^59^. In addition, the present study underlines the importance of rigorous control of covariates when investigating peripheral inflammatory markers.

An important and novel finding of the current study is the SZ specific negative correlation between composite cognitive score and plasma QUIN levels, an effect that remains significant after adjusting for several covariates. Cognitive deficits are a hallmark symptom of schizophrenia and have detrimental effects on functional outcome and quality of life^60,61^. While the underlying pathophysiological mechanisms are not well understood, cognitive dysfunctions have been associated with dysfunctional dopaminergic and glutamatergic neurotransmission in the prefrontal cortex and hippocampus^62^. QUIN was shown to be neurotoxic by inducing excitotoxicity (QUIN is a glutamatergic agonist acting on N-methyl-D-aspartate receptors [NMDARs]), promoting oxidative stress and disrupting energy homeostasis^63^. Direct evidence of a role of QUIN in eliciting cognitive deficits comes from pre-clinical rodent studies. Injections of QUIN into the medial prefrontal cortex of mice induced cognitive deficits and impairments in behavioural flexibility^64^. While increased QUIN and associated neurological and cognitive function have been associated with several neurodegenerative disorders such as Alzheimer’s disease^65^ or Huntington’s disease^66^, studies investigating its role in cognitive deficits in psychiatric disorders are scarce. One post-mortem study has demonstrated fewer QUIN-immunoreactive microglia cells in the hippocampus in patients with SZ compared to HC^67^. While these results might be contradictory to the current findings at first sight, it has to be noted that the reduced microglial expression of QUIN may represent compensatory mechanisms, attenuating the oxidative stress^67^. Given the function of QUIN as an NMDAR agonist it can be hypothesized that QUIN contributes to the glutamate theory of schizophrenia, which postulates that several symptom dimensions of schizophrenia are a result of hypofunctional NMDARs that affect the downstream mesolimbic and mesocortical pathways^68^. However, further studies are needed to elucidate the exact mechanisms of how dysregulation of QUIN contributes to glutamatergic hypofunction.

Interestingly, we did not find an association between cognitive deficits and TRYCATs in MDD. This is in contrast to a recent study, where Zhou et al. reported an association between cognitive impairment and elevated serum KYN levels and the KYN/TRP ratio. However, the authors found this association only in female but not male patients and further, QUIN was not studied^69^. Our study population was not large enough to investigate female and male patients separately and therefore future studies are needed to investigate potential sex differences in TRYCATs and cognitive deficits across neuropsychiatric disorders.

Contrary to our initial hypothesis, we did not observe an association between motivational deficits and TRYCATs in the SZ or MDD group. The rational for testing this hypothesis came from the observation that inflammatory processes in general and TRYCATs specifically can alter dopaminergic signalling, and the dopaminergic system plays an important role in negative symptoms and motivational deficits^15,70^. In addition, several pre-clinical studies have causally linked activation of the kynurenine pathway with several behaviours reminiscent of impairments in goal-directed behaviours^19,71^. However, the findings of the present study suggest that TRYCATs might be involved in other symptom dimensions than motivation.

There are strengths and limitations of the study that should be considered. The strengths are the well characterized patient population, the inclusion of both patients with SZ and MDD and the comprehensiveness of the TRYCATs measured. In addition, we only measured TRYCATs in the circulation but not in the central nervous system. Only KYN and 3-OHK can readily cross the BBB, while for example KYNA and QUIN access the brain via passive diffusion at a relatively low rate^15,72^. However, both pre-clinical studies in rodents and human neuroimaging studies provide some evidence that the BBB is impaired in neuropsychiatric disorders and thus certain neurotoxic substances that under physiological conditions do not cross the BBB could potentially access the brain in these conditions^73–76^. In addition, several studies comparing plasma with cerebrospinal fluid TRYCAT levels have revealed a high correlation, suggesting that peripheral TRYCAT levels indeed reflect levels in the central nervous system^77,78^. Further studies need to elucidate, if peripheral TRYCATs can access the brain facilitated by a disrupted BBB or if immune activation induces increased brain tryptophan catabolism independently from circulation, e.g. by infiltrating cytokines that activate microglia. One further limitation of the present study was the relatively small sample size of the control group resulting in limited power to detect small effects. In addition, motivational deficits were assessed using a clinician-rated scale and thus should in future studies be complemented by objective task based measures. Lastly, although the BNA is a well validated tool to assess cognitive function in patients with schizophrenia and has been shown to correlate well with other more comprehensive batteries of cognitive testing^35^, it will be important to follow up on the current findings with instruments that will allow for more detailed assessment of specific neurocognitive domains such as attention, working memory or executive function. Furthermore, although the two cognitive tests that constitute the BNA, the Letter-Number span (sequencing) test and the Symbol Coding Test are frequently used in depression research^79,80^, the BNA itself has not been validated in patients with MDD.

## Conclusions

In summary, the current study suggests that both MDD and SZ are associated with dysregulation of the kynurenine pathway. However, certain catabolites might contribute to different psychopathologies in specific disorders, e.g. QA might be involved in cognitive impairments in patients with SZ but not MDD. Further studies are needed to determine whether TRYCATs are causally involved in the etiology of these neuropsychiatric disorders and to elucidate the underlying mechanisms.

## Data Availability

The datasets of the current study are available from the corresponding author on reasonable request.
